# NEMS With Broken T Symmetry: Graphene Based Unidirectional Acoustic Transmission Lines

**DOI:** 10.1038/srep09926

**Published:** 2015-05-20

**Authors:** Mehdi B. Zanjani, Arthur R. Davoyan, Nader Engheta, Jennifer R. Lukes

**Affiliations:** 1 Department of Mechanical Engineering and Applied Mechanics, University of Pennsylvania, Philadelphia, PA 19104, USA; 2 Department of Electrical and Systems Engineering, University of Pennsylvania, Philadelphia, PA 19104, USA

## Abstract

In this work we discuss the idea of one-way acoustic signal isolation in low dimensional nanoelectromechanical oscillators. We report a theoretical study showing that one-way conversion between in-phase and anti-phase vibrational modes of a double layer graphene nanoribbon is achieved by introducing spatio-temporal modulation of system properties. The required modulation length in order to reach full conversion between the two modes is subsequently calculated. Generalization of the method beyond graphene nanoribbons and realization of a NEMS signal isolator are also discussed.

Low dimensional materials such as graphene^1^,^2^,^3^,^4^, carbon nanotubes^5^, boron nitride nanomaterials^6^,^7^,^8^,^9^, and atomically thin 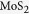
10 have attracted great interest in recent years. The extraordinary electrical^11^,^12^, optical^13^,^14^, thermal^15^,^16^, and mechanical^17^,^18^ properties of graphene and analogous low-dimensional materials^19^ make them promising candidates for practical applications in electronics, sensing, and energy storage devices.

Owing to their outstanding mechanical and electrical properties, these materials have been utilized as electromechanical oscillators in nanoscale memory cells and nanoelectromechanical switches^20^,^21^,^22^,^23^ and resonators^24^,^25^,^26^.The study of mechanical waves^27^ and the ability to manipulate, control, and detect vibrational motion in such nanoelectromechanical systems (NEMS)^28^,^29^,^30^,^31^ provides unprecedented opportunities to employ them in fluidic^32^, electronic^33^, and optical networks^34^. NEMS based oscillators as mechanical sensors and actuators are also used in applications such as ultrasensitive force^35^ and displacement detection^36^,^37^, scanning probe microscopy^38^ and resonant mass sensing of chemical and biological species^39^,^40^ where an important functionality is to have one-way communication channels that transmit desired signals only in one direction.

Acoustic rectifiers and diodes^41^,^42^,^43^,^44^,^45^ as well as one-way acoustic isolators^46^ have been recently studied. The acoustic diode concept is based on a nonlinear frequency conversion mechanism^44^,^47^,^48^. Other studies have focused on nonreciprocity in acoustic circulators and acoustic metamaterials^49^,^50^ as well as optomechanically induced nonreciprocity in resonators^51^,^52^. More recently, the idea of one-way phonon isolation^46^, motivated by optical equivalents^53^,^54^, was studied based on creating spatio-temporal modulation of mechanical properties. This leads to a one-way conversion between the guided modes, therefore breaking the symmetry of wave propagation in a waveguide in forward and backward directions.

In this letter we study the idea of one-way signal isolation in low dimensional nanoelectromechanical oscillators where the symmetry of the system under time reversal transformation, also known as the T-symmetry, is broken. To explain the method, we consider a system of graphene nanoribbons (GNRs) on an elastic substrate and demonstrate that the symmetry of wave propagation may be broken by introducing spatial and temporal modulation of elastic properties of the system. We show, both analytically and numerically, that in one of the propagation directions conversion between the modes occurs, whereas in the other direction the signal is transmitted without any perturbations. We also discuss the extension of this method beyond graphene nanoribbons and mention its possible implementation for designing a phonon isolator in nanoelectromechanical oscillators.

In Fig. 1(a), a double-layer graphene nanoribbon is shown. The system consists of two graphene nanoribbons with width b, each of which is perfectly adhered to an elastic substrate. We use nonlocal elasticity theory to study wave propagation along the nanoribbons^55^,^56^. In this model, shown in Fig. 2, each substrate is treated as a linear elastic medium with stiffness 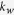
56, and the nanoribbons themselves interact via van der Waals forces that are also modeled as linear springs with stiffness 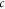
56. The governing equation for wave propagation in this system is driven from the nonlocal Euler-Bernoulli beam model^57^,^58^,
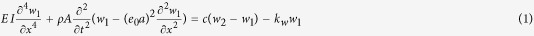

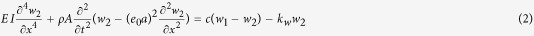
Here 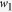 and 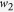 are flexural displacements of nanoribbons 1 and 2 in the y direction, 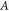 is the cross sectional area of each GNR, 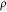 is the density, 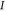 is the moment of inertia, 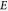 is the Young's modulus, 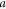 is the C-C bond length and 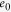 is a parameter representing nonlocal elastic effects in the GNR^56^,^57^. Without loss of generality, we consider a simplified case with 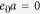 and 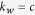
56, and use the parameters obtained in Ref^56^. The two governing equations (Eqs. 1 and 2) can then be written in the form 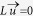 where 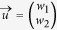, and 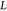 is a linear operator.

Solutions of the form 
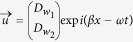
 are assumed, where 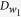 and 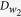 are the corresponding magnitudes of 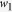 and 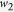, and 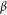 and 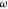 are the wavenumber and the angular frequency of the propagating wave. The corresponding solution results in two vibrational modes as shown in Fig 1(b). Mode 1 corresponds to the in-phase mode with 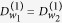 and mode 2 corresponds to the anti-phase mode with 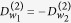. We note that below the cut-off frequency, 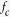, only the in-phase branch exists, see Fig. 1(b). The value of the cut-off frequency depends on the nanoribbon and elastic matrix properties^56^, 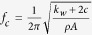, which in this system is around 20.5 THz for nanoribbons of 4 nm width^56^.

Clearly, the dispersion curves plotted in Fig. 1(b) are symmetric with respect to wavenumber 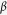, implying that the wave propagation in such a waveguide is reciprocal, i.e. waves traveling in forward and backward directions have the same properties. In order to break this symmetry in the wave propagation phenomenon we follow the technique suggested in Refs^46^,^53^. In particular, we consider wave propagation with a spatio-temporally modulated elastic matrix. In this case we expect that for an appropriately chosen modulation, one-way conversion between the guided modes may be induced, i.e. interaction between the guided waves is possible in one propagation direction only.

We assume spatio-temporal modulation of the elastic matrix constant so that 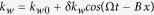 where 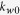 is the original stiffness constant and 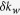 is the modulation depth. In order to maximize the coupling between modes, we modulate only the upper elastic matrix, as shown in Fig. 2. To solve the governing equations for the modulated system, we assume a general solution as the superposition of the guided modes: 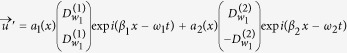, where 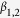 and 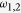 are the wavevectors and frequencies of in-phase and anti-phase modes, and 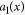 and 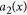 are their slowly varying spatial amplitudes. Next, using the standard techniques of the perturbation theory, two ordinary differential equations are obtained.
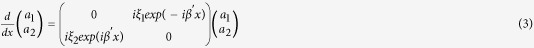
where 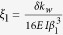, 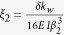, and 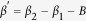. Based on the incoming signal frequency 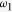 and the available modulation frequency 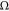, a full mode conversion from mode 1 to mode 2 occurs when 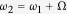 and when the 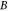 parameter of the modulation is chosen such that 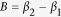 or 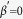. In this case, under phase-matching conditions, the differential equations can be simplified to
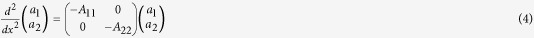
where 
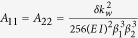
. The system shows one-way behavior because the modulation 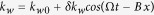 does not convert the mode at 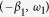 to any other modes. The resulting end point of the intended transition in the negative 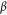 region does not lie on the anti-phase branch of the dispersion curve (Fig. 1(b)). This one-way behavior arises because the modulation breaks both time-reversal and spatial-inversion symmetry.

If we consider the propagation of the in-phase mode in the system, the initial conditions will be 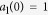 and 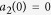. The solution to equation 4 is then written as 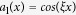 and 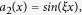 where 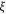 is the conversion wavevector. As described in Fig. 3(a), complete transition from in-phase to anti-phase mode is observed for the wave propagating in one direction (solid lines), while in the opposite direction the in-phase mode will not be influenced (dashed lines). The general solution of the governing equations is then 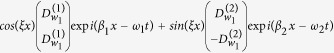 with 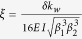, and the corresponding conversion length of 
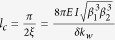
. As expected, the conversion length is inversely proportional to the modulation depth. This means that with stronger modulation, the mode conversion requires a smaller modulation domain. To analyze the conversion length further, we investigate its dependence on the modulation parameters. The intent of adding a modulation domain is to convert an incoming in-phase mode signal with frequency 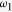 and wavenumber 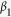 to the anti-phase mode using the available modulation frequency 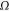 and tunable modulation wavenumber 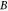. Therefore, we plot the dependence of the conversion length on wavenumber 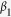 for different values of 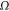. As shown in Fig. 3(b), the required conversion length is larger for higher wavenumbers, as 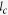 varies proportional to 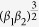 with 
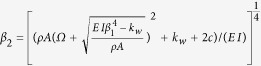
. It is worth mentioning that the choice of 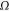 and 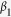 is not arbitrary and should satisfy two criteria. First, the corresponding mode 1 frequency, 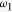, needs to be below the cut-off frequency. Second, the resulting frequency value 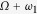 needs to lie on the dispersion curve of mode 2 in the forward propagation direction (positive 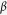 side) and lie on a band gap region in the backward propagation direction (negative 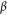 side) consistent with Fig. 1(b).

As an example, we choose a value slightly below the cut-off frequency to guarantee that at frequency 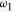 the system only supports the in-phase mode. For 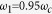, 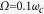 and 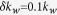, 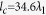 where 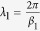 is the wavelength of the corresponding mode 1 signal. Here we use normalized quantities in both the analytical and the numerical calculations. The corresponding wavelength of mode 2 based on the dispersion curves will be 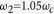. It is important to emphasize that the parameter values chosen are arbitrary and longer wavelength signals corresponding to smaller choices of 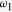 can also be used to observe the same isolation effect as long as the required conditions explained earlier are satisfied. Additionally, lower values of the modulation depth also result in a mode conversion, but require a longer modulation domain. Furthermore, the operational bandwidth is also important in evaluating the performance of the isolator. In order to estimate the bandwidth of the system operation depending on the modulation length, we have calculated the transmission characteristics of our system analytically. Typically, the outgoing signal can be a combination of mode 1 and mode 2 signals as described by the spatially varying mode amplitudes, 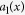 and 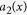, shown in Fig. 3(a). In the general case where a phase-mismatch could be present, the spatially varying amplitudes are given as 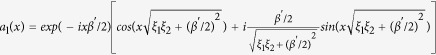 and 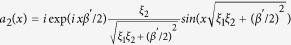, where 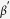 measures the phase mismatch introduced when the operational frequency 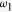 is changed by a small value, 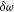. Since the modulation frequency and length are fixed, the frequency of the converted mode 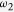 is also changed by the same value. The transmission coefficient of the converted mode can be calculated based on 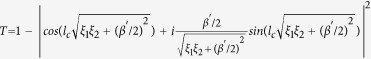. The resulting transmission coefficients are shown in Fig. 3(c) for a system with 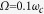, 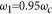, and with different modulation depths of 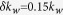, 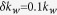, and 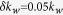, which correspond to modulation lengths of around 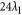, 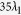 and 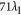 respectively. As shown in Fig. 3(c), the transmission coefficient drops fairly rapidly as the frequency is changed. This drop occurs because, due to the shape of the dispersion curves, the spatial modulation B is no longer commensurate with the difference between the wavenumbers of modes 2 and 1 at 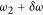 and 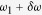. The corresponding bandwidth was calculated by considering a frequency span at which the transmission coefficient drops by 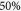. For the three cases studied here shown by the red, blue, and green curves, the bandwidth is around 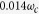, 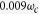, and 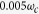 respectively. The results show that in order to have a larger operational bandwidth, a larger modulation depth needs to be provided.

In order to confirm the analytical model, we used numerical simulations of the wave propagation and mode conversion in double-layer GNRs using the finite difference time-domain (FDTD) method to solve the governing equations in the presence of spatio-temporal modulation. For this purpose, we choose a modulation domain with length 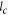 as predicted from the analytical model. The numerical simulation results are shown in Fig. 4. An incoming in-phase wave of wavelength 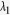 will be converted to an anti-phase wave of wavelength 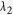 after passing through the modulation domain. The Fourier spectra of both the incoming and outgoing signals are shown in Fig. 4(b). From the FDTD simulations, the peak corresponding to the frequency of the outgoing signal (red curve) is around 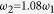 which shows good agreement with the analytical method. The design of the system provides conversion of a signal of mode 1 to a signal of mode 2 in only one of the propagation directions. Therefore, in the opposite direction where no conversion between the two modes happens, we essentially observe a transmission ratio of 1 for the signal. In the other direction the signal of mode 1 is converted to mode 2 which implies a transmission ratio of close to zero. In fact, qualitatively, this type of isolation results in the following scattering matrix for the two ports: 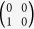. More specifically, if we consider the Fourier spectrum of the outgoing signal in Fig. 4(b), a transmission ratio of slightly above zero is observed for the forward propagation direction. However, this value of transmission ratio is influenced by the numerical errors within the framework of the FDTD method and it is important to keep in mind that the analytical method suggests a transmission ratio of zero for this scenario.

Experimental realization of spatio-temporal modulation of elastic properties has been investigated recently. Spatial property modulation is possible through periodic arrangement of elastic material similar to the case of phononic crystals^59^. Temporal property modulation, although more difficult, is also possible in practice through applying electric fields, magnetic fields or mechanical strains in a periodic fashion^60^,^61^,^62^. In general, spatial modulations with nanometer scale periodicity provide suitable conditions for conversion between the two vibrational modes of double-layer GNRs. Furthermore, THz range frequencies are appropriate for temporal modulations to guarantee the unidirectional nature of such systems. Beyond double-layer GNRs, the aforementioned technique is also applicable to other systems. The key here is to have a system with two different branches of dispersion curve separated by a cut-off frequency. Using the same method, one-way mode conversion is achievable in systems such as single layer GNRs or double-wall carbon nanotubes where similar types of governing equations describe their behavior^56^,^63^,^64^,^65^,^66^. It is also important to mention that for very short wavelength limits the physics of the problem may differ from the predictions of continuum models and therefore atomistic level techniques such as molecular dynamics simulations^67^ may be required for the analysis.

In this paper, we explored one-way acoustic signal isolation in graphene nanoribbons. We showed that through spatio-temporal modulation of the system properties, we can convert a signal of one mode to another mode in one direction, while no conversion is observed in the opposite direction. Combined with appropriate mode filters that filter out signals of frequency 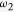, a signal of frequency 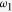 can be absorbed or filtered out after conversion to a signal of frequency 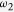 in one propagation direction while it will be transmitted with no disturbance as the same signal with frequency 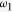 in the opposite direction. This method is not limited to graphene nanoribbons and can be used to induce the same type of signal isolation for acoustic wave propagation in other low dimensional oscillators. The realization of a NEMS based signal isolators raises intriguing possibilities for a wide range of applications in scanning probe microscopes, force and displacement detection devices and chemical and biological sensors.

## Author Contributions

M.B.Z. and A.R.D. performed the analytical and numerical calculations. J.R.L. and N.E. supervised the research. The manuscript was written through contributions of all authors.

## Additional Information

**How to cite this article**: Zanjani, M. B. *et al.* NEMS With Broken T Symmetry: Graphene Based Unidirectional Acoustic Transmission Lines. *Sci. Rep.* **5**, 9926; doi: 10.1038/srep09926 (2015).

## Figures and Tables

**Figure 1:**
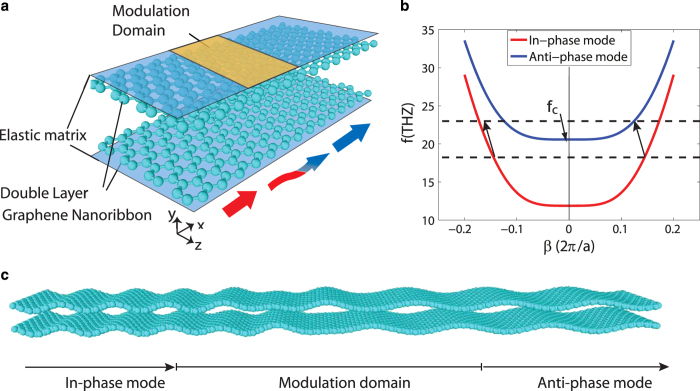
(**a**) A double-layer graphene nanoribbon on an elastic matrix. (**b**) Dispersion curve of the double-layer GNR obtained from Euler-Bernoulli beam model. The red and blue curves belong to the in-phase and anti-phase flexural modes, respectively. (**c**) Schematic of in-phase to anti-phase mode conversion in a double-layer GNR system.

**Figure 2:**
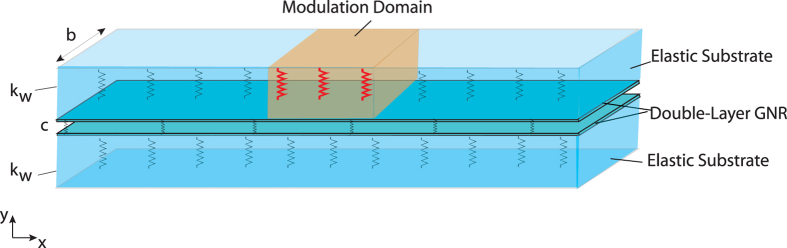
Schematic of continuum beam model for double-layer GNR on an elastic substrate with stiffness 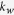. The shaded area represents the modulation domain.

**Figure 3:**
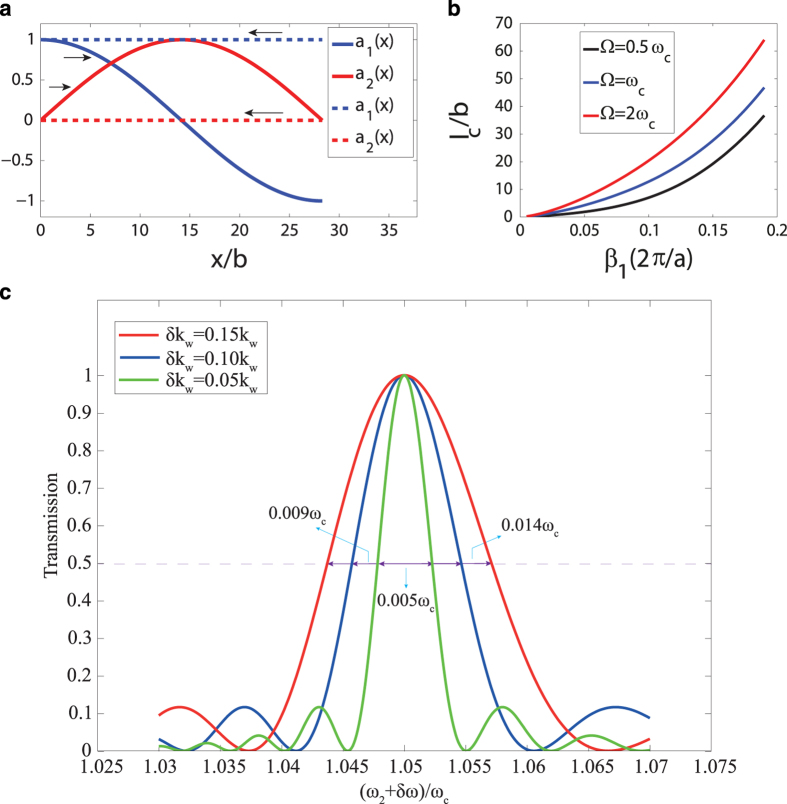
(**a**) Variation of mode amplitudes along the modulation domain. As shown by the arrows, the solid lines correspond to forward propagation with full conversion and the dashed lines correspond to backward propagation with no mode conversion. (**b**) Conversion length for different values of 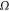 as a function of wave number of in-phase mode. (**c**) Transmission coefficient of mode 2 calculated for different operational frequencies. The red, blue, and green curves correspond to different modulation depths at 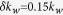, 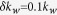, and 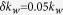, respectively. The bandwidths shown in the figure are determined by considering the frequency span for which the transmission coefficient drops by 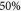.

**Figure 4:**
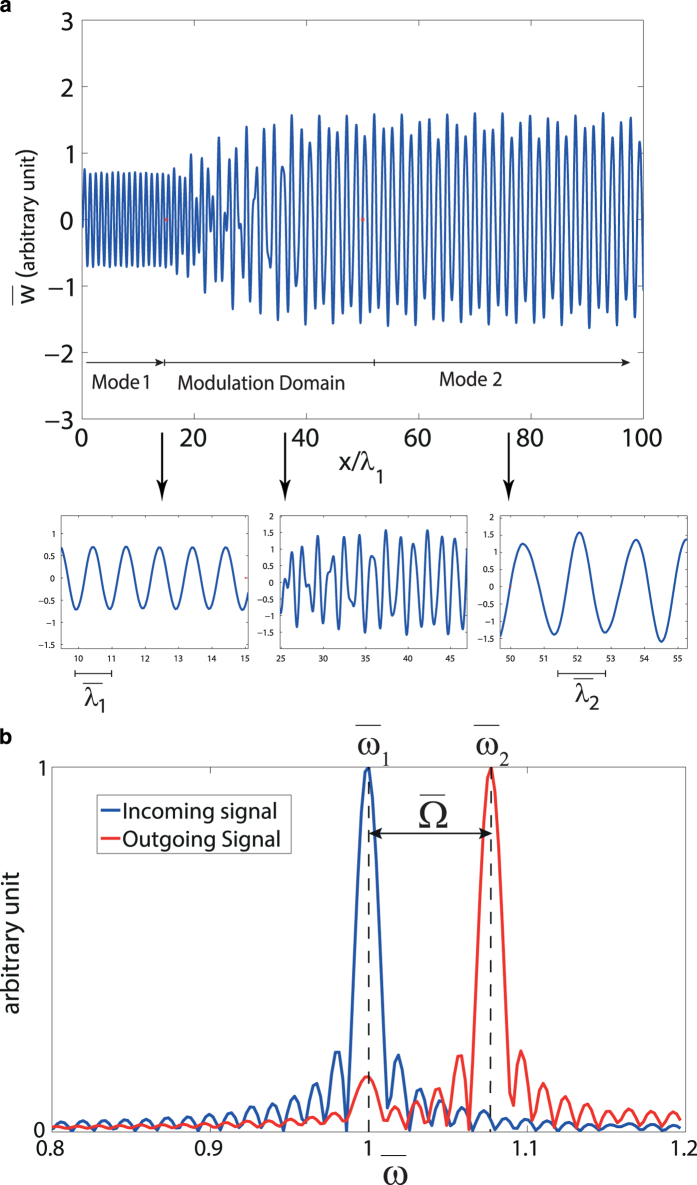
(**a**) FDTD simulation of in-phase to anti-phase mode conversion in double-layer graphene nanoribbons. x is scaled with respect to 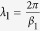. (**b**) Fourier spectrum of the signal before and after the modulation domain. The horizontal axis is normalized with respect to the frequency of mode 1. The blue curve shows the Fourier transform of mode 1 which is peaked at a value of 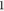 corresponding to 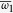. The red curve is the Fourier transform of the signal after the modulation domain which shows a peak at 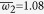.

## References

[b1] Novoselov K. S. *et al.* Electric field effect in atomically thin carbon films. Science 306, 666 (2004). 15499015 10.1126/science.1102896

[b2] Geim A. K. & Novoselov K. S. The rise of graphene. Nat. Mater. 6, 183 (2007). 17330084 10.1038/nmat1849

[b3] Meyer J. C. *et al.* The structure of suspended graphene sheets. Nature 446, 60 (2007). 17330039 10.1038/nature05545

[b4] Jia X. *et al.* Controlled formation of sharp zigzag and armchair edges in graphitic nanoribbons. Science 323, 1701 (2000). 19325109 10.1126/science.1166862

[b5] Bethune D. S. *et al.* Cobalt-catalysed growth of carbon nanotubes with single-atomic-layer walls. Nature 363, 605 (1993).

[b6] Chopra N. G. *et al.* Boron nitride nanotubes. Science 269, 966 (1995). 17807732 10.1126/science.269.5226.966

[b7] Chen Z.-G. *et al.* Novel boron nitride hollow nanoribbons. Science 2, 2183 (2008). 10.1021/nn8004922 19206466

[b8] Chen Y. J., Zhang H. Z. & Chen Y. Pure boron nitride nanowires produced from boron triiodide. Nanotechnology. 17, 786 (2006).

[b9] Suryavanshi A. P., Yu M.-F., Wen J., Tang C. & Bando Y. Elastic modulus and resonance behavior of boron nitride nanotubes Appl. Phys. Lett. 84, 2527 (2004).

[b10] Mak K. F., Lee C., Hone J., Shan J. & Heinz T. F. Atomically thin MoS2: A new direct-gap semiconductor. Phys. Rev. Lett. 105, 136805 (2010). 21230799 10.1103/PhysRevLett.105.136805

[b11] Zhang Y., Tan Y.-W., Stormer H. L. & Kim P. Experimental observation of the quantum Hall effect and Berry's phase in graphene. Nature 438, 201 (2005). 16281031 10.1038/nature04235

[b12] Wakabayashi K., Takane Y. & Sigrist M. Perfectly conducting channel and universality crossover in disordered graphene nanoribbons. Phys. Rev. Lett. 99, 036601 (2007). 17678303 10.1103/PhysRevLett.99.036601

[b13] Vakil A. & Engheta N. Transformation optics using graphene. Science 332, 1291 (2011). 21659598 10.1126/science.1202691

[b14] Liu M. *et al.* A graphene-based broadband optical modulator. Nature 474, 64 (2011). 21552277 10.1038/nature10067

[b15] Ghosh S. *et al.* Dimensional crossover of thermal transport in few-layer graphene. Nat. Mater. 9, 555 (2010). 20453845 10.1038/nmat2753

[b16] Balandin A. A. *et al.* Superior thermal conductivity of single-layer graphene. Nano Lett. 8, 902 (2008). 18284217 10.1021/nl0731872

[b17] Li D. & Kaner R. B. Graphene-based materials. *Science* 320, 1170 (2008). 10.1126/science.1158180 18511678

[b18] Lee C., Wei X., Kysar J. W. & Hone J. Measurement of the elastic properties and intrinsic strength of monolayer graphene. Science 321, 1170 (2008). 10.1126/science.1157996 18635798

[b19] Tang Q. & Zhou Z. Graphene-analogous low-dimensional materials. Progress in Materials Science 58, 1244 (2013).

[b20] Rueckes T. *et al.* Carbon nanotube-based nonvolatile random access memory for molecular computing. Science 289, 94 (2000). 10884232 10.1126/science.289.5476.94

[b21] Jang J. E. *et al.* Nanoscale memory cell based on a nanoelectromechanical switched capacitor. Nature Nanotechnol. 3, 26 (2008). 18654446 10.1038/nnano.2007.417

[b22] Stutzel E. U. *et al.* A graphene nanoribbon memory cell. Small 6, 2822 (2010). 20949540 10.1002/smll.201000854

[b23] Begliarbekov M., Strauf S. & Search C. P. Quantum inductance and high frequency oscillators in graphene nanoribbons. Nanotechnology 22, 165203 (2011). 21393820 10.1088/0957-4484/22/16/165203

[b24] Bunch J. S. *et al.* Electromechanical resonators from graphene sheets. Science 315, 490 (2007). 17255506 10.1126/science.1136836

[b25] Chen C. *et al.* Performance of monolayer graphene nanomechanical resonators with electrical readout. Nat. Nanotechnol. 4, 861 (2009). 19893525 10.1038/nnano.2009.267

[b26] Garcia-Sanchez D. *et al.* Imaging mechanical vibrations in suspended graphene sheets. Nano Lett. 8, 1399 (2008). 18402478 10.1021/nl080201h

[b27] Scarpa F., Chowdhury R., Kam K., Adhikari S. & Ruzzene M. Dynamics of mechanical waves in periodic graphene nanoribbon assemblies. Nanoscale Research Lett. 6, 430 (2011). 10.1186/1556-276X-6-430 PMC3211848 21711495

[b28] Craighead H. G. Nanoelectromechanical systems. Science 290, 1532 (2000). 11090343 10.1126/science.290.5496.1532

[b29] Verbridge S. S., Shapiro D. F., Craighead H. G. & Parpia J. M. Macroscopic tuning of nanomechanics: substrate bending for reversible control of frequency and quality factor of nanostring resonators. Nano Lett. 7, 1728 (2007). 17497822 10.1021/nl070716t

[b30] Papadakis S. J. *et al.* Resonant oscillators with carbon-nanotube torsion springs. Phys. Rev. Lett. 93, 146101 (2004). 15524813 10.1103/PhysRevLett.93.146101

[b31] Ilic B., Krylov S. & Craighead H. G. Theoretical and experimental investigation of optically driven nanoelectromechanical oscillators. J. Appl. Phys. 107, 034311 (2010).

[b32] Verbridge S. S., Bellan L. M., Parpia J. M. & Craighead H. G. Optically driven resonance of nanoscale flexural oscillators in liquid. Nano Lett. 6, 2109 (2006). 16968035 10.1021/nl061397t

[b33] Forsen E. *et al.* Ultrasensitive mass sensor fully integrated with complementary metal-oxide-semiconductor circuitry. Appl. Phys. Lett. 87, 043507 (2005).

[b34] Vlaminck I. D. *et al.* Detection of nanomechanical motion by evanescent light wave coupling. Appl. Phys. Lett. 90, 233116 (2007).

[b35] Mamin H. J. & Rugar D. Sub-attonewton force detection at millikelvin temperatures. Appl. Phys. Lett. 79, 3358 (2001).

[b36] Knobel R. G. & Cleland A. N. Nanometre-scale displacement sensing using a single electron transistor. Nature (London) 424, 291 (2003). 12867975 10.1038/nature01773

[b37] Yaralioglu G. G., Atalar A., Manalis S. R. & Quate C. F. Analysis and design of an interdigital cantilever as a displacement sensor. J. Appl. Phys. 83, 7405 (1998).

[b38] Manalis S. R., Minne S. C., Atalar A. & Quate C. F. Interdigital cantilevers for atomic force microscopy. Appl. Phys. Lett. 69, 3944 (1996).

[b39] Ilic B. *et al.* Enumeration of DNA molecules bound to a nanomechanical oscillator. Nano Lett. 5, 925 (2005). 15884896 10.1021/nl050456k

[b40] Ilic B. *et al.* Mechanical resonant immunospecific biological detector. Appl. Phys. Lett. 77, 450 (2000).

[b41] Liang B., Guo X. S., Tu J., Zhang D. & Cheng J. C. An acoustic rectifier. Nat. Mater. 9, 989 (2010). 20972430 10.1038/nmat2881

[b42] Boechler N., Theocharis G. & Daraio C. Bifurcation-based acoustic switching and rectification. Nat. Mater. 10, 665 (2011). 21785416 10.1038/nmat3072

[b43] Tanaka Y., Murai T. & Nishiguchi N. Rectification of elastic waves in a thin plate. J. Appl. Phys. 111, 024507 (2012).

[b44] Liang B., Yuan B. & Cheng J. C. Acoustic diode: Rectification of acoustic energy flux in one-dimensional systems. Phys. Rev. Lett. 103, 104301 (2009). 19792317 10.1103/PhysRevLett.103.104301

[b45] He Z. J. *et al.* Asymmetric acoustic gratings. Appl. Phys. Lett. 98, 083505 (2011).

[b46] Zanjani M. B., Davoyan A. R., Mahmoud A. M., Engheta N. & Lukes J. R. One-way phonon isolation in acoustic waveguides. Appl. Phys. Lett. 104, 081905 (2014).

[b47] Gu X., Lin Z., Liang B., Cheng J. & Zhang D. Modeling and optimization of an acoustic diode based on micro-bubble nonlinearity. J. Acoust. Soc. Am. 133, 1119–1125 (2013). 23363127 10.1121/1.4773256

[b48] Li X. F. *et al.* Tunable unidirectional sound propagation through a sonic-crystal-based acoustic diode. Phys. Rev. Lett. 106, 084301 (2011). 21405575 10.1103/PhysRevLett.106.084301

[b49] Fleury R., Sounas D. L., Sieck C. F., Haberman M. R. & Alu A. Sound isolation and giant linear nonreciprocity in a compact acoustic circulator. Science 343, 516 (2014). 24482477 10.1126/science.1246957

[b50] Popa B.-I. & Cummer S. A. Non-reciprocal and highly nonlinear active acoustic metamaterials. *Nat. Comm*. 5, 3398 (2014). 10.1038/ncomms4398 24572771

[b51] Hafezi M. & Rabl P. Optomechanically induced non-reciprocity in microring resonators. Opt. Express 20, 7672 (2012). 22453446 10.1364/OE.20.007672

[b52] Lenferink E. J., Wei G. & Stern N. P. Coherent optical non-reciprocity in axisymmetric resonators. Opt. Express 22, 16099 (2014). 24977863 10.1364/OE.22.016099

[b53] Yu Z. & Fan S. Complete optical isolation by indirect interband photonic transition. Nat. Photonics 3, 91 (2009).

[b54] Lira H., Yu Z., Fan S. & Lipson M. Electrically driven nonreciprocity induced by interband photonic transition on a silicon chip. Phys. Rev. Lett. 109, 033901 (2012). 22861851 10.1103/PhysRevLett.109.033901

[b55] Murmu T. & Pradhan S. C. Vibration analysis of nano-single-layered graphene sheets embedded in elastic medium based on nonlocal elasticity theory. J. Appl. Phys. 105, 064319 (2009).

[b56] Shi J., Ni Q., Li X. & Natsuki T. Wave propagation in embedded double-layer graphene nanoribbons as electromechanical oscillators. J. Appl. Phys. 110, 084321 (2011).

[b57] Allegri G., Scarpa F., Chowdhury R. & Adhikari S. Wave propagation in periodically supported nanoribbons: A nonlocal elasticity approach. J. Vibration and Acoustics 135, 041017 (2013).

[b58] Senalp A. D., Arikoglu A., Ozkol I. & Dogan V. Z. Dynamic response of a finite length euler-bernoulli beam on linear and nonlinear viscoelastic foundations to a concentrated moving force. J. Mech. Sci. Technol. 24, 1957 (2010).

[b59] Hopkins P. E. *et al.* Reduction in the thermal conductivity of single crystalline silicon by phononic crystal patterning. Nano Lett. 11, 107 (2011). 21105717 10.1021/nl102918q

[b60] Casadei F., Delpero T., Bergamini A., Ermanni P. & Ruzzene M. Piezoelectric resonator arrays for tunable acoustic waveguides and metamaterials. J. Appl. Phys. 112, 064902 (2012).

[b61] Cullen J. R., Rinaldi S. & Blessing G. V. Elastic versus magnetoelastic anisotropy in rare earthiron alloy. J. Appl. Phys. 49, 1960 (1978).

[b62] Jang J.-H., Ullal C. K., Gorishnyy T., Tsukruk V. V. & Thoma E. L. Mechanically tunable three-dimensional elastomeric network/air structures via interference lithography. Nano Lett. 6, 740 (2006). 16608275 10.1021/nl052577q

[b63] Bhaskar A. Elastic waves in Timoshenko beams: the 'lost and found' of an eigenmode. Proc. R. Soc. A 465, 239 (2009).

[b64] Yoon J., Ru C. Q. & Mioduchowski A. Vibration of an embedded multiwall carbon nanotube. *Compos. Sci. Technol*. 63, 1533 (2003).

[b65] Wang Q. Wave propagation in carbon nanotubes via nonlocal continuum mechanics. J. Appl. Phys. 98, 124301 (2005).

[b66] Natsuki T., Hayashi T., & Endo M. Wave propagation of carbon nanotubes embedded in an elastic medium. J. Appl. Phys. 97, 044307 (2005).

[b67] Allen M. P. & Tildesley D. J. Computer Simulation of Liquids (Oxford Science Publication 2001).

